# First trimester human umbilical cord perivascular cells (HUCPVC) modulate the kynurenine pathway and glutamate neurotransmission in an LPS-induced mouse model of neuroinflammation

**DOI:** 10.1186/s12950-023-00340-3

**Published:** 2023-05-01

**Authors:** Fyyaz Siddiqui, Denis Gallagher, Hannah Shuster-Hyman, Lianet Lopez, Andrée Gauthier-Fisher, Clifford L Librach

**Affiliations:** 1 CReATe Fertility Centre, 790 Bay Street, Suite 1100, Toronto, ON M5G 1N8 Canada; 2 Department of Obstetrics and Gynecology, Toronto, Canada; 3 Institute of Medical Sciences, University of Toronto, Toronto, ON Canada; 4 Department of Physiology, University of Toronto, Toronto, ON Canada

**Keywords:** Kynurenine pathway, Neuroinflammation, Mesenchymal stromal cells, HUCPVC

## Abstract

**Background:**

The Kynurenine Pathway (KP) of tryptophan degradation and glutamate toxicity is implicated in several neurological disorders, including depression. The therapeutic potential of mesenchymal stromal cells (MSC), owing to their well documented phagocytosis-driven mechanism of immunomodulation and neuroprotection, has been tested in many neurological disorders. However, their potential to influence KP and the glutamatergic system has not yet been investigated. Hence, this study sought to investigate the effect of HUCPVC, a rich and potent source of MSC, on Lipopolysaccharide (LPS)-activated KP metabolites, KP enzymes, and key components of glutamate neurotransmission.

**Methods:**

The immunomodulatory effect of peripherally administered HUCPVC on the expression profile of kynurenine pathway metabolites and enzymes was assessed in the plasma and brain of mice treated with LPS using LCMS and QPCR. An assessment of the glutamatergic system, including selected receptors, transporters and related proteins was also conducted by QPCR, immunohistochemistry and Western blot.

**Results:**

HUCPVC were found to modulate LPS-induced activation of KP enzymes and metabolites in the brain associated with neurotoxicity. Moreover, the reduced expression of the glutamatergic components due to LPS was also found to be significantly improved by HUCPVC.

**Conclusions:**

The immunomodulatory properties of HUCPVC appear to confer neuroprotection, at least in part, through their ability to modulate the KP in the brain. This KP modulation enhances neuroprotective regulators and downregulates neurotoxic consequences, including glutamate neurotoxicity, which is associated with neuroinflammation and depressive behavior.

**Supplementary Information:**

The online version contains supplementary material available at 10.1186/s12950-023-00340-3.

## Introduction

Mounting evidence has supported a direct and positive relationship between immune-activated proinflammatory cytokines and psychiatric disorders [1–3]. Pro-inflammatory cytokines elicited during systemic infection, cancer, and autoimmune diseases have been shown to play a significant role in the manifestations and propagation of a broad spectrum of depressive symptoms [4, 5]. Signaling of these peripherally originated proinflammatory cytokines to the brain through multiple pathways is implicated in neuroinflammation, perturbation of neurotransmitters’ metabolism, transport and function, and induction of depressive symptoms [6–9]. One such pathway that is activated by the immune response is the kynurenine pathway, which has been shown to play a key role in the activation of the central nervous system (CNS) and the pathogenesis of neuroinflammation and depression [10, 11].

**Fig. 1:**
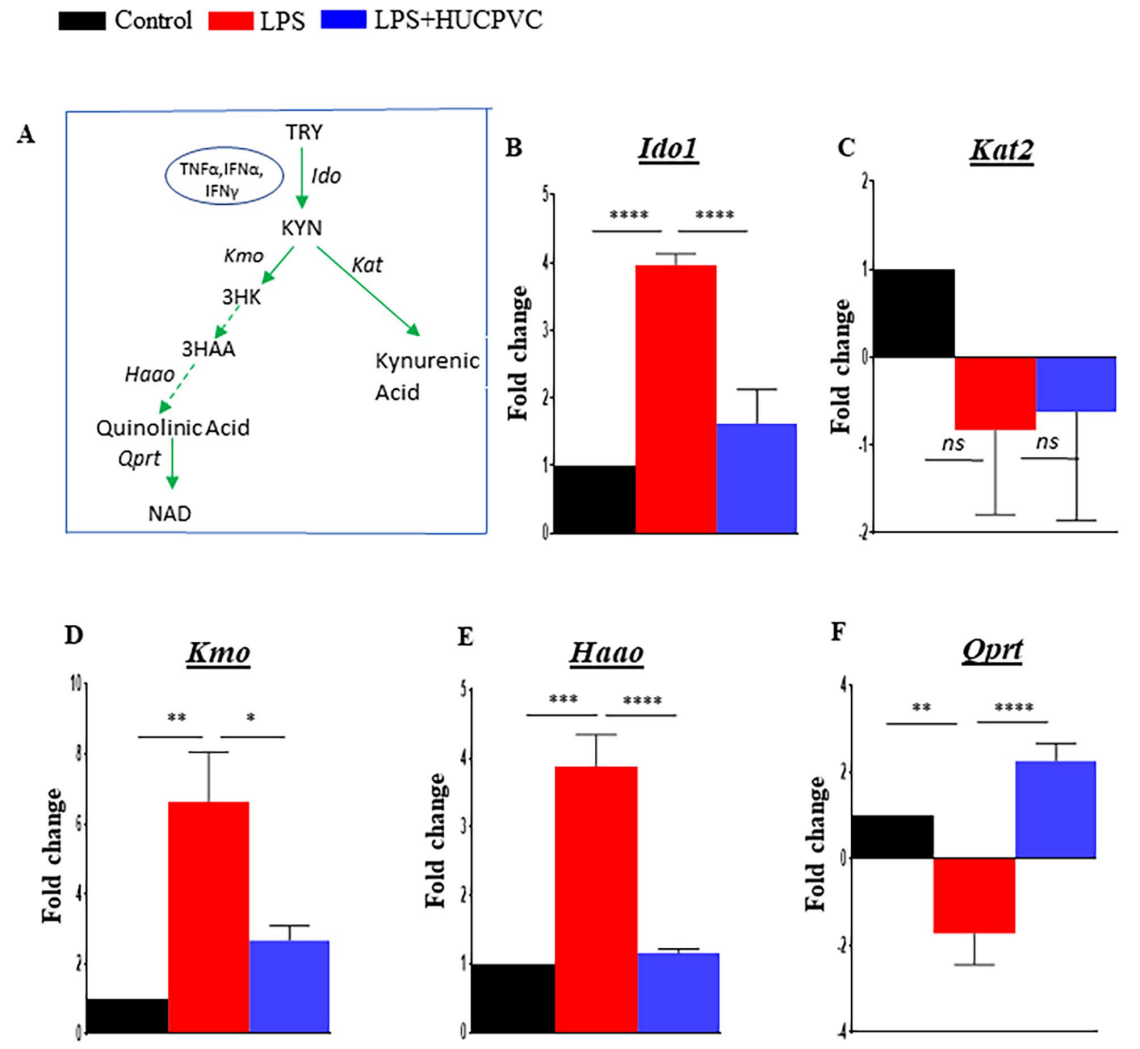
HUCPVC modulates LPS-induced activation of Kynurenine pathway enzymes Schematic diagram of the Kynurenine pathway (**A**). mRNA expression of Kynurenine pathway enzymes after 24 h LPS and HUCPVC treatments (**B**-**F**). Total RNA from the brain was isolated from Control, LPS, and LPS + HUCPVC groups (n = 3 per group) and subjected to qPCR. Individual measurements were normalized using GAPDH as a housekeeping gene and the Fold change relative to Control was calculated by the ΔΔCt method. *p < 0.05, **p < 0.005, ***p < 0.0005, ****p < 0.0001, *ns* = not significant, one-way ANOVA with Tukey’s multiple comparison tests. TRY, tryptophan; *ido*, indoleamine dioxygenase; KYN, kynurenine; *kat*, kynurenine aminotransferase; *kmo*, kynurenine monooxygenase; 3HK, 3-hydroxykynurenine; 3HAA, 3-hydroxyanthranilic acid; *haao*, 3-hydroxyanthranilic acid dioxygenase; *qprt*, quinolinate phosphoribosyltransferase; NAD, Nicotinamide adenine dinucleotide

The kynurenine pathway (KP) (Fig. 1A) claims the major share of tryptophan metabolism for the production of kynurenine and subsequent metabolites, while a meager amount of ingested tryptophan is converted to serotonin by the methoxyindole pathway [12, 13]. When induced by inflammatory signals, the extrahepatically expressed enzyme, indoleamine 2,3- dioxygenase (IDO), triggers activation of the kynurenine pathway by oxidatively breaking down tryptophan to kynurenine. IDO expressed in the brain contributes to centrally produced kynurenine which is further augmented by peripheral kynurenine crossing the blood-brain barrier [14]. Downstream catabolism of kynurenine takes two distinct routes leading to the production of excitatory and anti-excitatory metabolites- notably, quinolinic acid (QUIN) and Kynurenic acid (KYNA) which are a functional agonist and antagonist to the glutamate receptor- N-methyl-D-aspartate (NMDAR), respectively [15–17]. The association of these compounds with the glutamate receptor and extracellular glutamate availability, renders neuroprotective or neurotoxic character to these distinct routes of KP and their constitutive metabolites.

Dysregulation of KP resulting in a perturbation in the synthesis of neuroactive metabolites, notably KYNA and QUIN, have been associated with a plethora of neurodegenerative diseases and psychiatric disorders, including depressive illness [18]. Pharmacological interventions include the development of analogs of KYNA and inhibitors of neurotoxic enzymes that lead to the production of QUIN [18–20]. Mesenchymal Stromal Cells (MSC), owing to their capabilities to regulate the immune and inflammatory response have been an attractive therapeutic candidate for treating various diseases with inflammatory components [21]. However, their potential to modulate the host IDO activity and the downstream kynurenine pathway has not been tested.

We have previously demonstrated the efficacy of MSC in mitigating neuroinflammation and depressive behavior in both stress-induced, as well as LPS-induced preclinical models of depression and, have also outlined a phagocytosis-driven immunomodulation mechanism [22, 23]. In the current study, we aim to investigate the immunomodulatory and neuroprotective capabilities of a young source of MSC, HUCPVC, isolated from the perivascular region of a first trimester human umbilical cord, through a potential impact on kynurenine pathway modulation in an LPS-based mouse model of neuroinflammation; which to our knowledge has never been explored.

Glutamate excitotoxicity is implicated in many neurodegenerative diseases and psychiatric disorders [24, 25]. By their direct action on the glutamate receptors, QUIN and KYNA not only influence excitatory neurotransmission but also play an active role in the uptake and release of glutamate [26, 27]. Moreover, glutamate-induced excitotoxicity is directly related to a compromised glutamate transport system, that is associated with neuropathological conditions, including depression [28]. Thus, this study further investigates the possible role of MSC in the modulation of key glutamatergic neurotransmission components, including expression profiles of glutamate receptors (NMDA), glutamate transporters, and synaptosomal proteins.

## Materials and methods

### Animal treatment & cells

Previously established and characterized, pathogen-free lines of first trimester HUCPVC were used for this study, with ethics approval by the University of Toronto REB (#28,889) and by an independent accredited ethics board (VERITAS, #2576) [22]. 7–8-week-old male C57BL/6J mice were obtained from Charles River (Laval, Quebec). After arrival, mice were group-housed in standard shoebox cages, habituated for a week, and allowed ad libitum food and water access. General health was monitored daily by veterinary technicians or research staff.

MSC lines from human first-trimester umbilical cords were established and maintained as described previously [29]. As a control, human fibroblasts (HS-68 foreskin-derived) were purchased from ATCC (Manassas, VA). All cells were grown using minimum essential media with alpha modification, 10% fetal bovine serum, and 1% penicillin/streptomycin purchased from Gibco (Gaithersburg, MD). A fresh Solution of LPS (serotype O111:B4, New England Biolabs (Whitby, ON) was prepared on the day of injections by dissolving the compound in a sterile endotoxin-free isotonic saline. LPS (0.83 mg/kg) was administered intraperitoneally (i.p) in the LPS group. This dose is known to induce a full spectrum of the acute sickness response [30] and robustly increase IDO activity in the brain after 24 h– one of the mechanisms associated with LPS-induced depressive behavior in mice [31]. 1 × 10^6^ HUCPVC or HS68 cells resuspended in 200 µl of saline were injected intravenously (i.v) simultaneously with LPS in the LPS + HUCPVC or LPS + HS68 groups, respectively. 24 h after LPS treatment and cell or media injections, the mice were sacrificed for blood and whole brain collection. Untreated animals were included as a control group.

### RNA extraction, reverse transcription & real-time qPCR

Total RNA from whole-brain samples was extracted using Qiagen’s RNeasy Mini Kit (catalog# 74,104), according to the manufacturer’s protocol. The eluted RNA was quantified using Nanodrop (NanoVue, GE). 1 µg of total RNA was used for reverse transcriptase reactions that were carried out in a Verity 96-well Thermal cycler (Applied Biosystems, model# 9902), using the High-Capacity cDNA Reverse Transcription kit (Thermo Fisher Scientific, catalog# 4,368,814), according to the manufacturer’s protocol. Real-time qPCR was performed for 40 cycles on a QuantStudio5 thermocycler (Applied Biosystems). Primers used were *Gapdh* (NM_008084), *Ido1* (NM_008324), *Kat2* (NM_011834), *Kmo* (NM_133809), *Haao* (NM_025325), *Qprt* (NM_133686.1), *NR1* (NM_008169.3). *NR2A* (NM_008170.4), *NR2B* (NM_008171.4). GAPDH was used as an endogenous reference gene for the normalization of mRNA levels and relative quantification of gene expression. Fold change from the qPCR data was measured by the delta-delta Ct method.

### Immunohistochemistry, image analysis & quantification

Mice were euthanized by cervical dislocation and trans-cardially perfused with cold PBS followed by 4% paraformaldehyde (PFA). Brains were harvested and fixed in 4% PFA for 24 h at 4 °C. Cryopreservation was done by immersing the harvested brains in 30% sucrose for 1–2 days, followed by snap-freezing in optimum cutting temperature (OCT) formulation (Electron Microscopy Sciences). Serial coronal tissue sections were obtained at a 16–18 μm thickness on a cryostat at TCP (the Centre for Phenogenomics, Toronto). For each animal (n = 3 animals per group) 6–8 sections were processed for immunohistochemistry. After briefly rinsing with PBS, the sections were fixed in 4% PFA for 15 min and blocked with 5% Normal Goat or Donkey serum (Sigma) supplemented with 1% BSA (Sigma), and 0.4% Triton-X-100 (Fisher) in PBS, for 1–2 h at room temperature. Tissue sections were then incubated overnight at 4 °C with the primary antibodies in 50% diluted blocking solution, followed by 1 h incubation with suitable fluorescently labeled secondary antibodies (Alexa Fluor1:1000) at room temperature. For immunostaining of nuclei, the sections were incubated with DAPI for 5 min before mounting the slides with coverslips using mounting media (Abcam). Primary antibodies used were: Mouse monoclonal anti-GFAP antibody [2A5] (1:200, Abcam, catalog# ab4648); rabbit monoclonal EAAT2 [E3P5K] (1:50, Cell Signalling Technology, catalog# 20,848); anti-IBA1 (1:1000, Wako Chemicals, Richmond, VA). Confocal microscopy was performed using a Leica TCS SP8 confocal microscope at the advanced optical microscopy facility (AOMF), University Health Network, Toronto. The confocal microscope was equipped with fully spectral 400 to 700 nm filters and HyD high-sensitivity detectors. Images were processed using Leica LAS-X software. 6–9 representative images were taken at x20 magnification spanning the cerebral cortex and hippocampus. Image thresholds and tissue surface area marked by positive staining were analyzed using ImageJ software. For quantification of EAAT2 and GFAP expression, the mean percent area of EAAT2 positive staining was normalized with that of GFAP for a given field of view for all images across different experimental groups.

### Western blotting

#### Whole-brain homogenization

Brains were harvested and immediately snap-frozen in liquid nitrogen and stored at -80 °C. Brain tissues were later placed in RIPA buffer (Sigma, catalog # 0278) in 2ml silica tubes prefilled with 3 mm zirconium beads and subjected to high-velocity impact homogenization using the BeadBug 6, Six Position Homogenizer (Benchmark Scientific, catalog # D1036). Homogenization was done at 3000 rpm for 30 s followed by 30 s on ice-, repeating the cycle 3 times. The homogenate was then centrifuged at 12,000 rpm for 15 min at 4 °C and the supernatant was collected in the pre-chilled tubes.

#### Synaptosome protein fractionation

Synaptic protein extraction was performed following the protocol from Thermo Scientific. Briefly, freshly harvested whole brains, excluding the cerebellum (200-400 mg), were homogenized in 10 volumes of the Syn-PER synaptic protein extraction reagent (Thermo Fisher; catalog# 87,793) using a Dounce tissue grinder, performing 10–12 up-and-down strokes. The homogenate was then centrifuged at 1200 g for 10 min to remove cell debris, and the supernatant was centrifuged at 15,000 g for 20 min. The pellets, containing synaptosomes, were gently resuspended in 1–2 ml of the Syn-PER reagent.

The resulting whole-brain homogenate/synaptosomal protein lysate was assayed for protein concentration using a BCA protein assay kit (Thermo Scientific, catalog# 23,225), and stored at − 80 °C. These protein samples were placed in a reducing buffer containing β-mercaptoethanol and heated at 90 °C for 10 min. Samples were then subjected to SDS–polyacrylamide gel electrophoresis in 4–12% Tris-Glycine 1 mm precast gels (Invitrogen, catalog# XP04120BOX), and then transferred to polyvinylidene fluoride membranes using a semi-wet Mini Blot Module transfer unit (Life Technologies, catalog# B1000). The membranes were blocked in LiCor blocking buffer for 1 h at room temperature and probed with the primary antibodies in 0.1% Tween LiCor blocking buffer overnight at 4 °C. The next day, the membranes were washed 3 times for 10 min each in 0.01% tween phosphate buffer solution before probing with an appropriate mix of IR-Dye-labeled Licor secondary antibodies in 0.1% Tween, 0.01% SDS LiCor blocking buffer for 1 h at room temperature. Washes, as described above, were repeated after incubation with secondary antibodies. Western blot images were obtained on a Licor Odyssey Imaging System. Relative quantification of the protein bands was assessed by densitometry analysis using ImageJ software. Primary antibodies used were: rabbit monoclonal EAAT2 [E3P5K] (1:1000, Cell Signalling Technology, catalog# 20,848); rabbit monoclonal anti PSD95 antibody [EPR23124-118] (1:2000, Abcam, catalog# ab238135); mouse monoclonal anti NMDAR2B/NR2B (1:500, Invitrogen, catalog# MA-1-2014), rabbit monoclonal anti Drebrin antibody [EPR12634] (1:10,000, Abcam, catalog# ab178408) and rabbit monoclonal anti Synaptophysin antibody [YE269] (1:10,000, Abcam, catalog# ab32127).

### Extraction and quantification of brain and plasma samples - LCMS Analysis

Sample extraction & preparation, LC-MS/MS analysis, and quantification were performed by the Analytical Facility for Bioactive Molecules (AFBM), Hospital for Sick Children, Toronto, Canada.

All LC-MS/MS grade solvents were purchased from Caledon Laboratories Ltd (Georgetown, ON). Autosampler vials/glass inserts used in the sample extraction were purchased from Chromatographic Specialties Ltd (Brockville, ON).

150–180 mg of frozen brain tissues were weighed and transferred into Precellys homogenization tubes containing ceramic beads (Bertin Technologies, Rockville, Washington DC). The entire brain was processed for this assay which required two tubes per sample depending on the brain weight. Tissue samples were kept overnight in the − 80 °C freezer until extraction. The following day, extraction solvent was added to each Precellys tube to achieve a target concentration of 150 mg/mL and homogenized using a Precellys 24 high-throughput homogenizer (Bertin Technologies). Plasma (100 µL) and brain samples (67 µL of the homogenized suspensions (corresponding to 10 mg tissues) were transferred into Eppendorf tubes containing 1 ml 90:10 acetonitrile (ACN):methanol (MeOH) alongside standards, quality control standards, and deuterated internal standards. Tubes were vortexed and then centrifuged at 20,000 g. Supernatants were transferred to a conical tube and taken to dryness under a gentle stream of nitrogen. Samples were reconstituted in 90/10 H_2_O/ACN + 0.1% formic acid and analyzed by LC/MS/MS.

An Agilent 1200 UPLC system (Agilent Technologies, Santa Clara, CA, USA) fitted with a Sciex Q-Trap 5500 mass spectrometer (AB Sciex, Framingham, MA, USA) was used in Electron Spray Ionization (ESI) mode. Two methods were employed. Except for a few analytes, most analytes were quantified using a Kinetex PFP column (2.6 μm, 100Å, 50 × 3.0 mm; Phenomenex, Torrence, CA). A gradient mobile phase of 10 min at a flow rate of 0.4 ml/min was used for the elution of the biogenic amines with mobile phase A (MPA): 1% acetic acid in water and mobile phase B (MPB): 1% acetic acid in 1:1 MeOH:ACN. For the remainder of the analytes, an EZ Fast 4uaaa-MS column was used. A gradient mobile phase of 10 min at a flow rate of 0.4 ml/min was used with MPA (0.1% formic acid, 0.1% heptafluorobutyric acid in water) and MPB (0.1% formic acid in MeOH). Quantification was performed with Analyst 1.6.1 software (ABSciex: Framingham, Massachusetts, USA) by plotting the sample peak area ratios (Analyte peak area/Internal Standard peak area) of the biogenic amine standards against a standard curve generated from various standard concentrations from 0.05 ng to 100 ng, spiked with the same amount of internal standard used for the samples and extracted using the same conditions.

### Statistical analysis

All data were represented as mean ± SEM and analyzed using Ordinary one-way analysis of variance followed by post hoc pairwise Tukey’s multiple comparisons tests comparing all the experimental groups using GraphPad Prism (GraphPad Software, San Diego, CA).

## Results

### Peripheral HUCPVC infusion modulates LPS-induced activation of kynurenine pathway enzymes in the brain

Proinflammatory cytokines mediate the induction of IDO which serves as a molecular switch for triggering the initiation of the KP [32]. Enzymatic activity and expression of IDO, which is also present in brain endothelial cells, perivascular macrophages, astrocytes, and microglia [33], have a direct influence on brain tryptophan metabolism [34]. Our previous study demonstrated significant mitigation of LPS-induced proinflammatory cytokines in the brain by peripherally administered HUCPVC in mice [23]. Thus, we sought to investigate the impact of the immunomodulatory potential of HUCPVC on the gene expression profile of *Ido1* and the subsequent metabolic enzymes of the pathway in the LPS-activated CNS (Fig. 1A). Our results indicate significant induction of *Ido1* in the brain by LPS (p < 0.0001) and its equally significant downregulation back to control levels as a result of HUCPVC treatment (p < 0.0001) (Fig. 1B). *Ido*-mediated synthesis of kynurenine is centrally placed in the KP as it is further favorably catabolized by the enzymes *Kmo* and *Kat* towards putative neurotoxic or neuroprotective branches, respectively [19]. In the LPS group, we recorded no significant changes in mRNA levels of *Kat2*, when compared to control (p = 0.2949) or LPS + HUCPVC (p = 0.9846) groups (Fig. 1C). Conversely, a significant LPS-induced upregulation of *Kmo* (p = 0.0020) was seen to be modulated back to near control levels by HUCPVC (p = 0.0176) (Fig. 1D). The excitatory properties associated with the ability of quinolinic acid to stimulate NMDA receptors directly and selectively are well known [17]. This neurotoxic compound is synthesized by the enzymatic activity of *Haao.* We observed that LPS significantly increased the expression of *Haao* (p = 0.0002) and that the treatment of LPS combined with HUCPVC helped downregulate its level significantly (p = 0.0001) (Fig. 1E). Accumulation of toxic concentrations of quinolinic acid also depends on the rate of metabolism of quinolinic acid to NAD + by the enzyme *Qprt.* Our results indicate that the immune response to LPS resulted in a significant lowering of *Qprt* expression (p = 0.0033) when compared to the control group and LPS + HUCPVC effected a significant upregulation of the enzyme transcript (p < 0.0001) (Fig. 1F). This data suggests that the influence of HUCPVC treatment on *Qprt* expression may be instrumental in regulating the synthesis of KP metabolites.

### Influence of HUCPVC on LPS-activated kynurenine pathway metabolites and serotonin in the brain and plasma

**Fig. 2:**
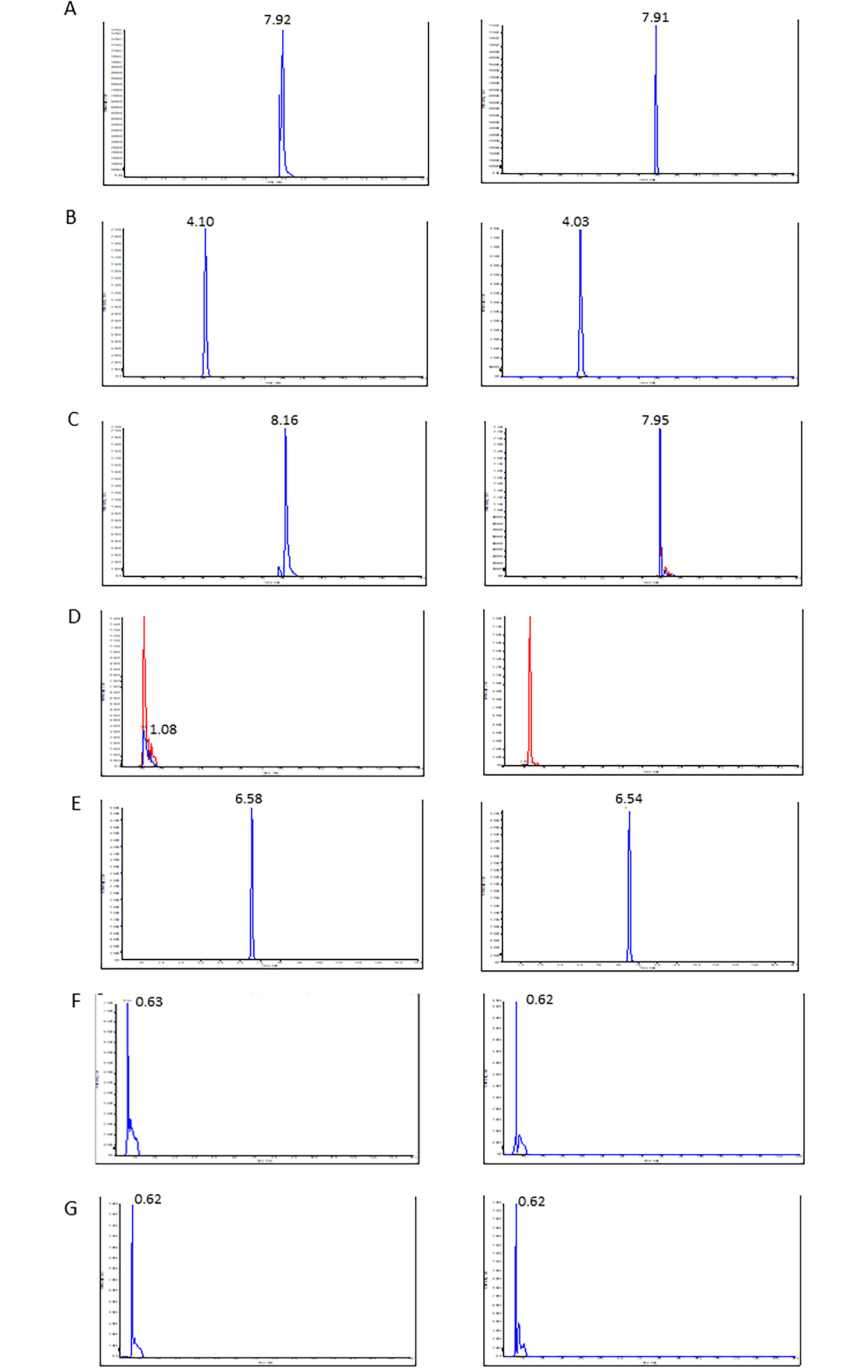
Chromatograms of kynurenine pathway metabolites Tryptophan, **B**) Kynurenine, **C**) Kynurenic acid, **D**) Quinolinic acid, **E**) Serotonin, **F**) Glutamine, **G**) Glutamate. Peak area ratios for the standards and the corresponding analytes are shown on the left and right sides respectively (Peak intensity on the y-axis and retention time on the x-axis of the chromatograms)

The induction of KP metabolites following 24 h of LPS treatment and intervention by HUCPVC were assessed by LC-MS/MS. The standard curves were linear over the concentration ranges. The calibration curves were as follows: y = 0.166 x + 0.166, R^2^ = 0.9922 for tryptophan; y = 0.349x + 0.0344, R^2^ = 0.9860 for kynurenine; y = 0.0943x + 0.000927, R^2^ = 0.9934 for kynurenic acid; y = 0.00715x + 0.0175, R^2^ = 0.9857 for quinolinic acid; y = 0.196x + 0.00148, R^2^ = 0.9930 for serotonin; y = 1.12e-005x + -5.35e-005, R^2^ = 0.9989 for glutamine and y = 3.67e-005x + -0.00039, R^2^ = 0.9973 for glutamate. The representative chromatograms of these analytes are shown in Fig. 2A-G. As detailed in Table 1, LPS significantly upregulated brain levels of tryptophan (p = 0.0197), kynurenine (p < 0.0001), kynurenine:tryptophan (p < 0.0001) and quinolinic acid (p = 0.0003), when compared to control. No significant difference was found in the levels of kynurenic acid (p = 0.9503) or serotonin (p = 1417). However, the neurotoxic index, quinolinic acid:kynurenic acid was found to be significantly increased by LPS (p = 0.0117), when compared to control. The combination of LPS with HUCPVC significantly modulated the brain levels of tryptophan (p = 0.0217), kynurenine (p = 0.0390), kynurenine:tryptophan (p = 0.0401) and quinolinic acid (p = 0.0042) when compared to LPS alone. No significant difference was found in the levels of kynurenic acid (p = 0.9598) or serotonin (p = 0.7892). However, quinolinic acid:kynurenic acid was found to be significantly increased in this condition (p = 0.0149). Since brain kynurenine levels are directly impacted by peripheral circulation [13], plasma tryptophan metabolism was also assessed (Table 1). No significant effect of LPS was found in the plasma levels of tryptophan (p > 0.9999), kynurenine (p = 0.2420), quinolinic acid (p = 0.9409), kynurenic acid (p = 0.6114), quinolinic acid:kynurenic acid (p = 0.7065) or serotonin (p = 0.9999) when compared to control. However, the IDO activation index, Kynurenin:tryptophan was found to be significantly increased by LPS (p = 0.0320) when compared to control. No significant modulation was noted by HUCPVC on plasma levels of tryptophan (p = 0.9930), kynurenine (p > 0.9999), kynurenine:tryptophan (p = 0.8341), kynurenic acid (p > 0.9999), quinolinic acid (p = 0.4393), quinolinic acid:kynurenic acid (p = 0.7698) or serotonin (p = 0.9978) when compared to LPS-treated animals. The immunomodulatory effect of a control non-MSC cell type was tested by using human foreskin-derived fibroblasts (HS68) in lieu of HUCPVC, and their influence on LPS-activated KP metabolites was assessed. There was no significant effect on brain levels of tryptophan (p = 0.2513), kynurenine (p = 0.2901), kynurenic acid (p > 0.9999), quinolinic acid (p = 0.9980), quinolinic acid:kynurenic acid (p = 0.9998) or serotonin (p = 0.9984) when compared to LPS-treated animals. However, a significant effect was recorded for kynurenine:tryptophan (p = 0.0035). Moreover, HS68 was found to have no significant effect on the plasma levels of tryptophan (p = 0.9991), kynurenine (p = 0.7195), kynurenine:tryptophan (p = 0.4029), kynurenic acid (p = 0.9485), quinolinic acid (p = 0.9752), quinolinic acid:kynurenic acid (p = 0.8721) or serotonin (p = 0.8125). These experimental read-outs support the possibility that peripherally infused HUCPVC have an influence on the KP metabolism in the brain.

**Table 1:** TRY, tryptophan; KYN, kynurenine; KYNA, kynurenic acid; QUIN, quinolinic acid; 5-HT, serotonin; Glu, glutamate; Gln, glutamine, LPS, lipopolysaccharide; HUCPVC, human umbilical cord perivascular cells; HS68, human foreskin fibroblast cells. Data represents mean (± SEM). n = 5–8 mice per group, (except HS68, n = 3). ^a^Significant effect of LPS (versus Control), ^b^significant effect of HUCPVC (versus LPS), ^c^significant effect of HS68 (versus LPS), Ordinary One-way ANOVA with Tukey’s multiple comparison test. *p < 0.05, **p < 0.005, ***p < 0.0005, ****p < 0.0001 Assessment of Kynurenine pathway metabolites, serotonin, glutamate and glutamine levels by LC-MS after 24 h LPS treatment along with either HUCPVCs or HS68

Metabolite	Control	LPS	LPS + HUCPVC	LPS + HS68
***Brain***				
TRY (nM)	172 (8.09)	209 (7.28)^a*^	170 (7.16)^b*^	236 (17.1)
KYN (nM)	12.8 (0.96)	61.1 (3.46)^a****^	43 (5.97)^b*^	47.9 (9.93)
KYN:TRY	0.077 (0.009)	0.338 (0.01)^a****^	0.238 (0.025)^b*^	0.173 (0.079)^c**^
KYNA (nM)	1.257 (0.155)	1.388 (0.060)	1.513 (0.237)	1.39 (0.206)
QUIN (nM)	3044 (604)	7892 (362)^a***^	4086 (651)^b**^	8090 (1311)
QUIN:KYNA	3081 (511)	5717 (330)^a*^	3080 (676)^b*^	5790 (229)
5-HT (nM)	385 (40.9)	520 (50.6)	464 (42.6)	507 (3.33)
Glu (µM)	909 (66)	893 (36)	893 (43)	1103 (281)
Gln (µM)	1853 (179.5)	2025 (162.1)	2012 (138.4)	2374 (583)
***Plasma***				
TRY (µM)	0.758 (0.030)	0.756 (0.033)	0.741 (0.046)	0.746 (0.103)
KYN (µM)	0.824 (0.061)	1.155 (0.168)	1.155 (0.133)	0.915 (0.040)
KYN:TRY	1.106 (0.103)	1.71 (0.212)^a*^	1.54 (0.104)	1.28 (0.208)
KYNA (µM)	0.159 (0.005)	0.14 (0.014)	0.141 (0.010)	0.151 (0.003)
QUIN (µM)	4.389 (0.392)	4.751 (0.507)	3.808 (0.465)	4.403 (0.336)
QUIN:KYNA	27.9 (3.16)	44.3 (17.7)	30.1 (4.38)	29.2 (2.75)
5-HT (µM)	3.19 (0.411)	3.24 (0.525)	3.12 (0.494)	2.47 (0.070)
Glu (µM)	11.1 (1.82)	12 (2.19)	9.31 (1.88)	10.1 (3.12)
Gln (µM)	170.1 (26.6)	169.5 (24.4)	135.1 (18.1)	176.5 (20.5)

Since the above data suggested the modulation of LPS-induced kynurenine metabolites was the result of HUCPVC intervention, the non-MSC control HS68 treatment group was, thus, excluded from the following experiments.

### Impact of peripherally administered HUCPVC on LPS-induced neuroinflammation and glutamatergic neurotransmission

#### Microglial activation by LPS and modulation by HUCPVC

**Fig. 3:**
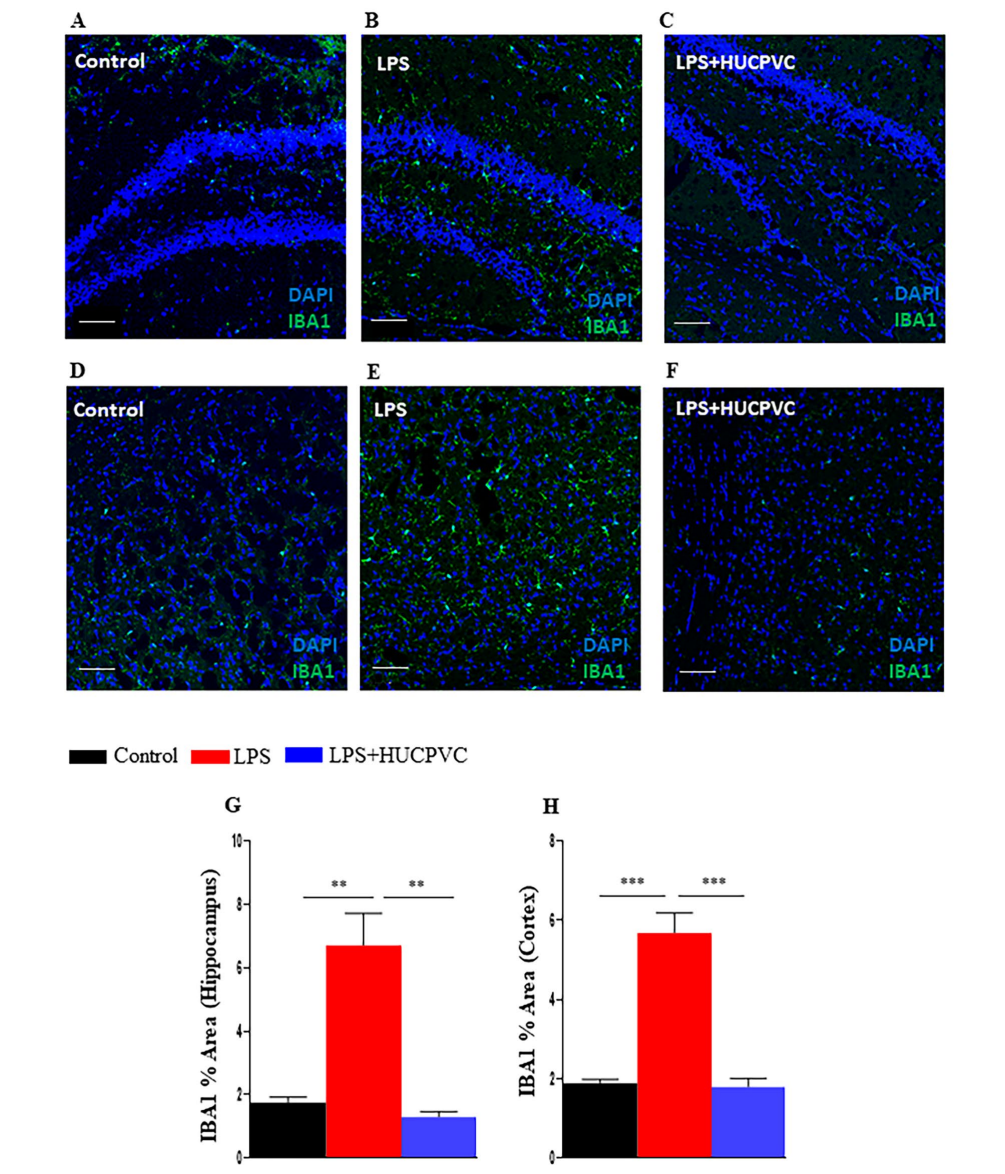
HUCPVC modulate LPS-induced microglial activation. Representative images showing positive immunostaining of microglial activation marker, ionized calcium-binding adapter molecule 1 IBA1 (green), in the hippocampus (**A,B,C**) and cortex (**D,E,F**) in the brain cryosections of control (**A,D**), LPS (**B,E**) and LPS + HUCPVC (**C,F**) groups. Nuclei counterstained with DAPI (blue). Scale bar = 50 μm. The mean percent area of IBA1 positive signal in the hippocampus (**G**) and cortex (**H**) was assessed by Image J analysis, *n* = 3 per group. *p < 0.05, **p < 0.005, ***p < 0.0005, one-way ANOVA with Tukey’s multiple comparison tests

Corroborating our previous study where we reported significant mitigation of LPS-induced proinflammatory cytokines in the brain by peripherally administered HUCPVC [23], in this study we demonstrate the potential of HUCPVC potential to modulate neuroinflammation by measuring LPS-induced microglial activation in the hippocampus (Fig. 3A,B,C**)** and cortex (Fig. 3D,E,F), LPS significantly increased activation of microglia as assessed by increased positive staining for ionized calcium-binding adapter molecule 1 (IBA1) in the dentate gyrus region of the hippocampus (p = 0.0028) (Fig. 3G) as well as the cortex (p = 0.0003) (Fig. 3H). Significant modulation of this induction by HUCPVC was seen both in the dentate gyrus (p = 0.0018) (Fig. 3G) as well as the cortex (p = 0.0003) (Fig. 3H).

#### The regulation of glutamate transporters & receptors by HUCPVC infusion

**Fig. 4:**
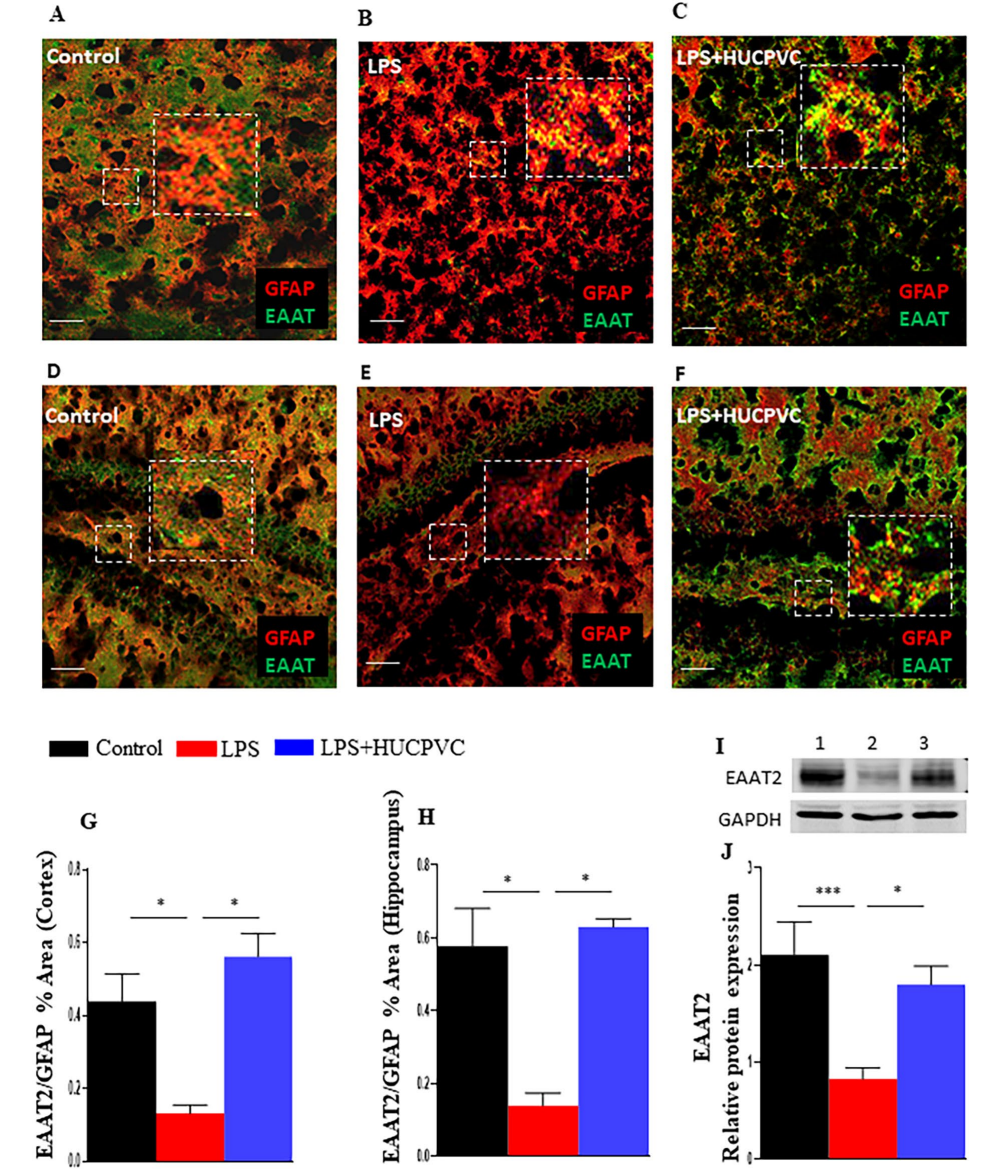
HUCPVC rescues the LPS-induced decline of glutamate transporter in the brain Representative images showing double staining of astrocyte marker glial fibrillary acidic protein (GFAP, red) and excitatory amino acid transporter 2 (EAAT2, green) in the cerebral cortex region (**A,B,C**) and dentate gyrus region of the hippocampus (**D,E,F**) in the brain cryosections of control (**A,D**), LPS (**B,E**), and LPS + HUCPVC (**C,F**) groups. Scale bar = 50 μm. Immunohistochemistry data for cortex (**G**) and hippocampus (**H**) represented as the ratio of mean percent area of EAAT2 and the corresponding GFAP positive signals of a given field of view for each section was assessed by Image J analysis, *n* = 3–5 per group. Representative Western blot of EAAT2 (**I**) expression in Control (1), LPS (2) & LPS + HUCPVC (3) groups. Densitometric analysis of EAAT2 protein expression (**J**). Data normalized to GAPDH, n = 5–9 per group. *p < 0.05, **p < 0.005, ***p < 0.0005, one-way ANOVA with Tukey’s multiple comparison tests

**Fig. 5:**
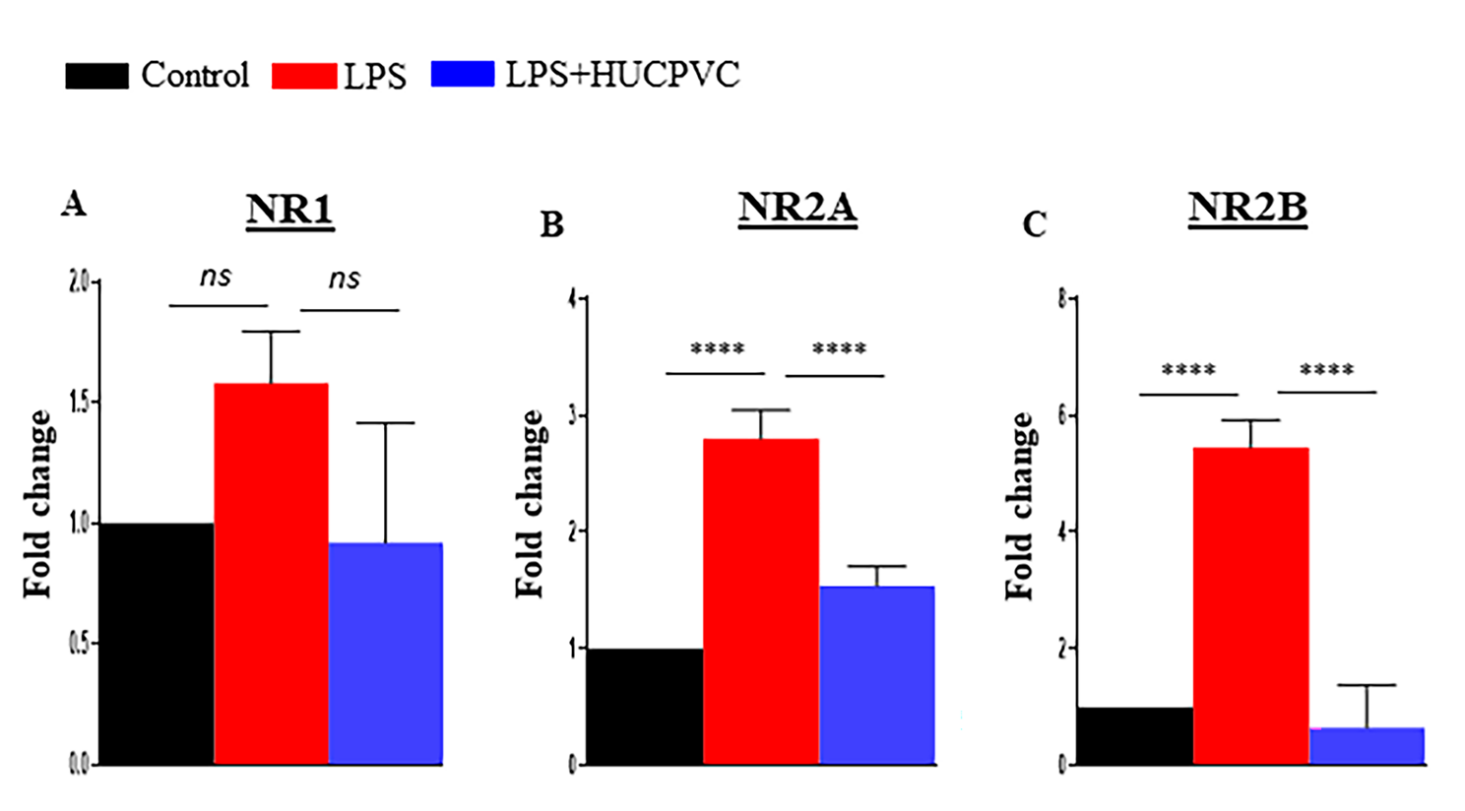
HUCPVC-mediated modulation of NMDAR in the brain. mRNA expression levels of glutamate receptor, NMDAR subunits, NR1 (**A**), NR2A (**B**) and NR2B (**C**) in Control, LPS and LPS + HUCPVC groups. Fold change calculated by ΔΔCt method. Data normalized to GAPDH, n = 3 per group, *p < 0.05, **p < 0.005, ***p < 0.0005, ****p < 0.0001, *ns* = not significant, one-way ANOVA with Tukey’s multiple comparison tests

Glutamate-mediated excitotoxicity is linked to various neurodegenerative diseases and psychiatric disorders [24, 35]. Since the synaptic levels of glutamate are predominantly maintained by excitatory amino acid transporters (EAATs), we tested the expression of EAAT2 in the brain in response to peripheral immune activation and HUCPVC infusion. In this experiment, EAAT2 was co-stained with glial fibrillary acidic protein (GFAP), a marker for astrocytes, where it is primarily localized [36]. Overall EAAT2 expression was assessed by normalizing its positive signal with that of GFAP in the cerebral cortex and dentate gyrus (DG) region of the hippocampus. The results revealed that LPS significantly diminished EAAT2 expression levels in the cerebral cortex (p = 0.0319, Fig. 4B, G) and hippocampus (p = 0.0182, Fig. 4E, H). Conversely, LPS in combination with HUCPVC significantly reinstated the transporter’s expression level both in the cerebral cortex (p = 0.0102, Fig. 4C, G) and hippocampus (p = 0.0180, Fig. 4F, H). This differential EAAT2 protein expression in control, LPS and LPS + HUCPVC-treated animals was also demonstrated by Western blot. We observed an obvious loss of EAAT2 immunoreactivity due to LPS, which was regained close to the control level in the HUCPVC-treated group (Fig. 4I). The densitometric data analysis (Fig. 4J) confirmed significant downregulation of EAAT2 expression by LPS (p = 0.0010) and significant rescue by HUCPVC treatment (p = 0.0034). NMDAR is one of the prominent glutamate receptors that mediate the activity of glutamate and 2 main subunits, NR1 and NR2A/B, are obligatory for the receptor’s activity and signaling [37]. We tested the transcript levels of these NMDAR subunits in the mRNA isolated from whole brains of the control, LPS, and LPS + HUCPVC groups (Fig. 5A-C). LPS was found to significantly upregulate the transcript expression of *NR2A* (p < 0.0001) and *NR2B* (P < 0.0001). Conversely, HUCPVC significantly downregulated *NR2A* (P = 0.0006) and *NR2B* (p < 0.0001) transcripts. However, no significant change was observed in the *NR1* gene expression in both LPS (p = 0.4128) and LPS + HUCPVC (p = 0.3258) groups, when compared to control.

#### Extended role of HUCPVC in regulating synaptosomal proteins linked to glutamate trafficking & receptor activity

**Fig. 6:**
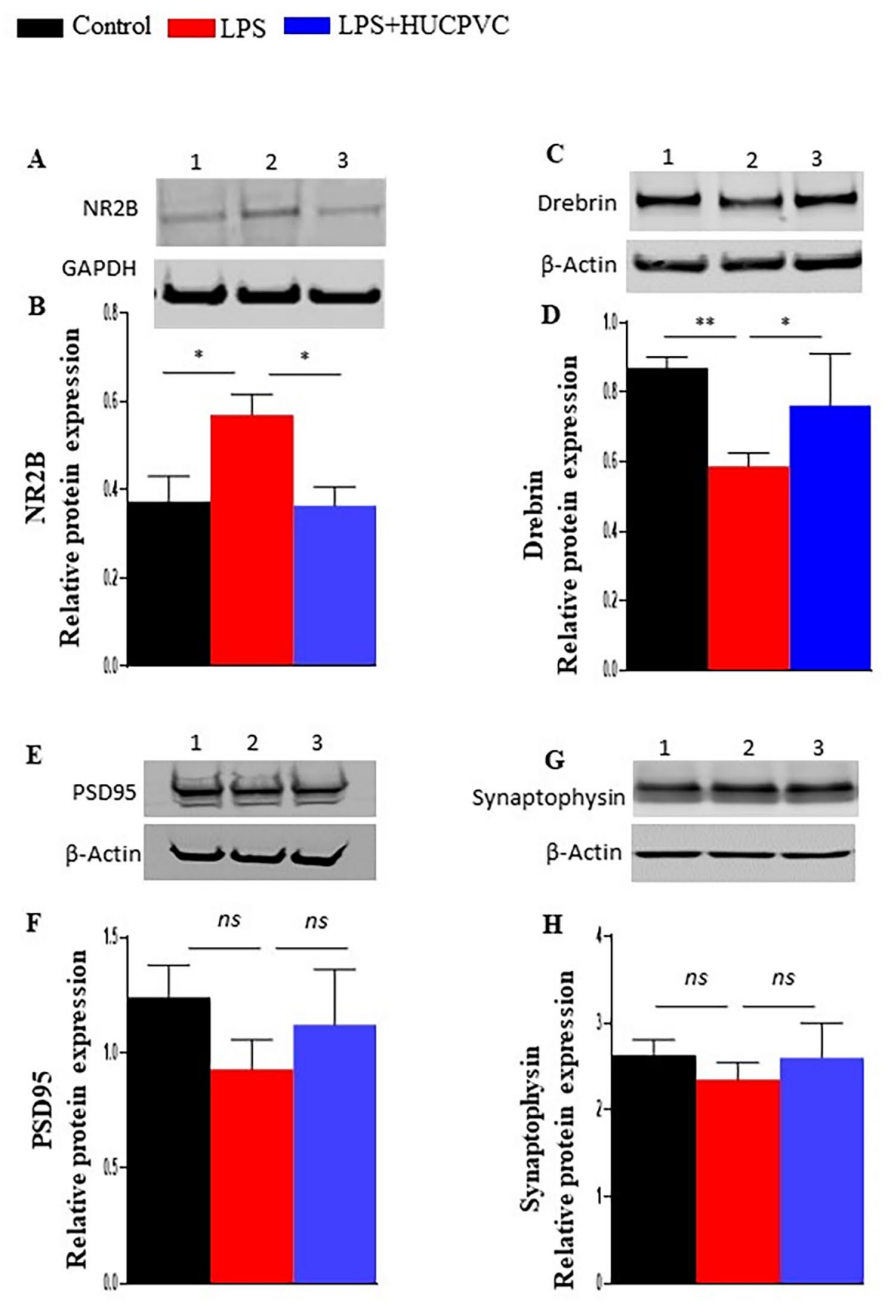
HUCPVC modulates the expression of synaptosomal proteins. Representative Western blots and relative protein expression of NR2B (**A, B**), Drebrin (**C, D**), PSD95 (**E, F**), and Synaptophysin (**G, H**) in Control (1), LPS (2) & LPS + HUCPVC (3) groups. The data is normalized to GAPDH or b-actin as mentioned, n = 6–9 per group, *p < 0.05, ‘*ns*’, not significant, one-way ANOVA with Tukey’s multiple comparison tests

The synaptosomal fraction is enriched with several proteins that impact glutamate receptor (NMDAR) activity and signaling. By Western blot, we tested some of these proteins such as NMDAR subunit- NR2B, postsynaptic density protein (PSD95), synaptophysin, and drebrin which are associated with glutamate trafficking and NMDAR function [38–40]. A significant increase in NR2B expression by LPS (p = 0.0386) was observed which was equally counteracted by HUCPVC intervention (p = 0.0299) (Fig. 6A, B). The expression level of drebrin was found to be significantly downregulated by LPS (p = 0.0013) and rescued by HUCPVC (p = 0.0386) (Fig. 6C, D). However, no change in the expressions of either PSD95 (Fig. 6C, D) or synaptophysin **(**Fig. 6E, F**)** was found to be induced by LPS alone or in combination with HUCPVC.

## Discussion

Exploiting the well-known immunomodulatory potential of MSC, this study is the first, to our knowledge, to examine the influence of intravenously infused MSC on the activated kynurenine pathway (KP) and glutamate neurotransmission. Here, we report that peripherally administered HUCPVC regulate KP enzymes and metabolites in the LPS-activated CNS. Furthermore, these MSC were also found to exert a modulatory effect on the expression profile of glutamate receptor subunits and glutamate transporters. Thus, this study lends a novel approach to target aberrant signaling of KP and the subsequent glutamate excitotoxicity that are known to have diverse neuropathological consequences.

Inflammation-associated upregulation and activation of IDO (product of *ido1* gene) in the brain is a critical step in the break down of tryptophan to kynurenine and initiation of the KP (Fig. 1A) [11, 41, 42]. Moreover, IDO activation is shown to be crucial for depressive-like behavior in mice treated with LPS for 24 h [11, 32]. We have previously reported that HUCPVC modulate neuroinflammation and depressive behavior after 24 h of LPS injection in mice [23]. In this study, we report that HUCPVC regulate *IDO* at the transcriptional level and the enzyme’s activation in the brain, as assessed by kynurenine:tryptophan ratio. This finding informs us of the possibility that the modulation of inflammation-associated depressive behavior by HUCPVC could be due to their ability to influence the catalytic function of the host IDO. Kynurenine synthesized by IDO can be a favorable substrate for either astrocytic enzyme KAT leading to the production of neuroprotective KYNA or microglial enzymes KMO and HAAO leading to the production of neurotoxic QUIN. Consistent with other studies [43, 44], we recorded an LPS-induced aberration in the brain mRNA levels of *Kmo, Haao* and *Kat2*, which is a predominant isomer of the KAT enzyme responsible for KYNA production [45]. Since the inadequate metabolism of QUIN by the enzyme QPRT contributes to increased neurotoxicity [46], we also tested the brain mRNA level of the enzyme, which was sharply downregulated by LPS. Thus, the recovery of an LPS-activated imbalance of the KP enzymes by HUCPVC treatment indicates the capability of MSC to influence the KP enzymatic machinery of glia governing the central production of KP metabolites.

KP metabolite levels in the brain have been intensely interrogated owing to their strong affiliation with many CNS disorders [1, 47]. In the current study, we evaluate the downstream immunomodulatory effect of intravenously administered HUCPVC on the neurotoxicity index, as assessed by the brain QUIN/KYNA ratio. Since the majority of brain kynurenine is peripherally derived during inflammation [13], plasma levels of the KP metabolites were also evaluated. Furthermore, to delineate the potential MSC-associated immunomodulatory effect, human foreskin-derived fibroblast cells (HS68), which have a relatively low level of immunomodulatory potential compared to MSC [48, 49], were independently injected into a group of animals. Our results indicate that compared to the brain, IDO activation in the plasma was not sufficient to increase the flux of circulating kynurenine. We hypothesize that this mild increase in kynurenine:tryptophan ratio, as shown in this study and by others [50], could merely be the statistical outcome of non-significant changes in tryptophan or kynurenine levels, which likely holds little metabolic or clinical significance. This lack of increase in plasma kynurenine may be due to its rapid clearance by the kidney and excretion of its metabolites in urine [51, 52]. Sufficient IDO-induced tryptophan oxidation is required to exceed the effect of renal processing and result in appreciable levels of plasma kynurenine. Moreover, kynurenine being a substrate to KMO, the inflammation-induced expression and/or activity of KMO can counter the effect of IDO. In addition, since circulating proinflammatory cytokines are shown to peak within 1–6 h of LPS treatment [40, 53], arguably, these early timepoints could correspond to a relatively higher plasma IDO activity, as shown by Wirthgen et al. [54]. Thus, the low plasma levels of other KP metabolites in the current study could be the downstream effect of this lack of increase in kynurenine. Conversely, in the brain, we noted significant aberration of KP metabolite levels due to LPS. Significantly altered KP metabolites in the brain due to LPS were rescued back to basal levels by HUCPVC treatment; an effect not seen for the most part with fibroblast cells. These findings reveal significant and specific immunomodulatory effect of MSC on KP metabolism in response to LPS; thus maintaining homeostasis between two functionally contradictory branches of the pathway. Interestingly, and in accord with other studies [55], we found an increase in tryptophan by LPS. This increase, which could be due to LPS-induced lipolysis resulting in increased availability of albumin-free tryptophan to cross the blood-brain barrier (BBB) [56], was reversed by HUCPVC. Cytokine-stimulated IDO activation is also known to have a negative impact on serotonin (5HT) turnover [57]. Since the level of 5-hydroxy indole acetic acid (5HIAA), the metabolite of 5HT was not examined in this study, the unchanged 5HT level does not reflect its actual turnover and thus does not preclude the possibility of inflammation-afflicted modulation of serotonergic neurotransmission.

The nexus between activated cerebral KP metabolism and NMDAR activation, leading to enhanced glutamate function, is associated with many neuropathological conditions [58]. The observed HUCPVC-induced modulation of KP metabolites in this study, notably that of QUIN, an endogenous NMDAR agonist, provoked further investigation into the expression profile of the obligatory subunits of NMDAR, that mediate the receptor’s activity, and whose upregulation is implicated in various brain pathologies, including inflammation-related depressive phenotype [59]. In this study, we report a significant increase in the transcript levels of the subunits, *NR2A* and *NR2B*, in response to LPS, which resonates with similar observations by others [60, 61]. The demonstrated ability of HUCPVC to modulate these subunits may suggest a novel and relatively safer therapeutic alternative to the NMDAR subunit-targeting antidepressants that are shown to have psychoactive side effects and cardiovascular toxicity [62]. Glutamatergic circuitry is also negatively impacted by the perturbed transport mechanism responsible for the clearance of synaptic glutamate, leading to glutamate excitotoxicity [28]. Our study, for the first time, illustrates the potential of MSC, specifically HUCPVC, in regaining the LPS-induced decline in the astrocytic glutamate transporter EAAT2. Since the levels of glutamate (Glu) and glutamine (Gln) in the blood and brain also reflect glutamate excitotoxicity [63], we tested these metabolites in the plasma and whole-brain homogenate by LCMS. However, levels of Glu and Gln showed no significant changes between the control and LPS treatment groups. Since Glu and Gln cross BBB [64] and are expressed differentially in different brain regions [65], the lack of modulation in Glu and Gln levels reported in this study could be the consequence of the limitations of the methodology, which is unable to distinguish between the source of the metabolites in the plasma (central vs. peripheral) or to delineate the region-specific expression of Glu and Gln in the brain. We further extended the scope of this study to investigate the immunomodulatory effect of systemically infused MSC on the neuroinflammation-associated synaptic imbalance implicated in neurodegenerative and psychiatric illnesses, including depression [66]. The effect of peripherally administered HUCPVC on LPS-induced dysregulation of synaptic markers such as drebrin, synaptophysin, PSD95, and NMDAR regulatory subunit NR2B, was tested in a synaptosomal isolate. Drebrin, an actin-binding protein in dendritic spines and one of the key players in the NMDAR-dependent synaptic neurotransmission, has been shown to be negatively regulated by the neuroinflammatory cascade associated with neurodegenerative diseases and psychiatric disorders [38, 67]. The HUCPVC-mediated restoration of LPS-induced downregulation of drebrin expression suggests a novel potential application of MSC in restoration of synaptic loss. Furthermore, we report the upregulation of NR2B expression in response to LPS. This corroborates the previous findings that the proinflammatory cytokines in the brain facilitate the activation of the NMDAR subunit [67]. We chose NR2B for the protein expression study as it is the most prominent of NMDAR subunits with respect to its synaptic signaling and its pharmacological suppression has been achieved using various selective antagonists in various brain pathologies [68]. However, these pharmacological agents cause undesirable side effects including neurotoxicity and hypertension [69]. Thus, our findings support the possibility that MSC may be a potentially safer and more efficient therapeutic alternative to target NR2B.

There has been a considerable lack of understanding of the basis of systemically administered MSC’s ability to provide neuroprotection in many diseases and injury models. In our previous study using an LPS-induced mouse model of neuroinflammation and depression, we have demonstrated phagocytosis-driven immunomodulation by peripherally infused HUCPVCs [23]. Here, the canonical mechanism of innate immune cascade was shown to be comprised of MSC-induced recruitment of neutrophils and their subsequent rapid clearance by recipient macrophages. The resulting systemic innate immune alteration from pro- to anti-inflammatory phenotype was plausibly correlated to the mitigation of LPS-induced neuroinflammatory response and depressive symptoms. Notably, considering the different routes of administration of the MSC and LPS and a short bioavailability of the MSC [23] we exclude the likelihood of cellular uptake of LPS and attribute the immunomodulatory potential of MSC in mediating LPS-induced immune response. Similar observation in our study related to stress-induced murine model of depression suggests the host immune cell-mediated phagocytosis of MSC trigger an immunomodulatory cascade, resulting in resolution of inflammation (22) In the current study, modulation of LPS-activated microglia via the kynurenine pathway in the CNS by HUCPVC may be another mechanism for peripheral immune modulation by the MSCs. Considering our previous findings that peripherally infused MSC fail to cross BBB [22], an alternate mechanism of MSC neuroprotective potential is its paracrine action, by which secreted factors are shown to mediate neuroprotection [70–72]. Contextually, studies showing the interplay between the kynurenine pathway and MSC support the involvement of IDO in the immunosuppressive effect of MSC [73]. Moreover, KYNA is also shown to regulate the expression of TNF-stimulated gene 6 (TSG-6), a paracrine factor, and promote TSG-6-mediated immunosuppressive and anti-inflammatory effects of MSC [74]. Yet another underlying mechanism for MSC-mediated skewing of macrophages toward anti-inflammatory M2 phenotype is recently reported [75]. Here, MSC-derived IDO-catalyzed kynurenine was shown to activate aryl hydrocarbon receptor (AhR) that enhanced binding to the promoter of nuclear factor (erythroid-derived 2)-like 2 (NRF2) in macrophages resulting in their polarization. However, the modulation of a broad spectrum of activated kynurenine metabolites and the downstream signaling consequences vis-à-vis the glutamatergic system by MSC, as shown in this study, represents a novel finding.

## Conclusion

This is the first study to examine and demonstrate the capability of intravenously injected MSC to regulate KP metabolites in a neuroinflammatory context, thus maintaining an optimal balance between ‘neuroprotective’ and ‘neurotoxic’ branches of the pathway. The novelty of this study is also marked by the demonstrated potential role of MSC in curbing glutamate excitotoxicity. In conclusion, our research findings further our knowledge to understand the mechanistic aspect of MSC neuroprotection, specifically through the KP. In addition, the specific anti-inflammatory properties we observed support the potential for use of MSC, particularly HUCPVC, as a therapeutic option for targeting inflammation-driven activated KP metabolites and glutamatergic systems linked to neurological diseases and affective disorders.

## Electronic supplementary material

Below is the link to the electronic supplementary material.

Supplementary Material 1

## Data Availability

The datasets used and/or analyzed during the current study are available from the corresponding author on reasonable request.

## Abbreviations

ACN: Acetonitrile
BBB: Blood-brain barrier
CNS: Central nervous system
DG: Dentate gyrus
EAAT: Excitatory amino acid transporter
GFAP: Glial fibrillary acidic protein
Gln: Glutamine
Glu: Glutamate
3HAA: 3-Hydroxy anthranilic acid
3HAAO: 3-Hydroxy anthranilic acid dioxygenase
5HIAA: 5-Hydroxy índole acetic acid
3HK: 3-Hydroxykynurenine
HUCPVC: Human Umbilical Cord Perivascular cells
IDO: Indoleamine 2,3- dioxygenase
IP: Intraperitoneal
IV: Intravenous
KAT: Kynurenine aminotransferase
KMO: Kynurenine monooxygenase
KP: Kynurenine Pathway
KYN: Kynurenine
KYNA: Kynurenic acid
LC-MS: Liquid Chromatography with tandem mass spectrometry
LPS: Lipopolysaccharide
MeOH: Methanol
MSC: Mesenchymal stromal cell
NAD: Nicotinamide adenine dinucleotide
NMDAR: N-methyl-D-aspartate receptor
OCT: Optimum cutting temperature
PFA: Paraformaldehyde
QPRT: Quinolinate phosphoribosyl transferase
QUIN: Quinolinic acid
5HT: Serotonin
TRY: Tryptophan
TSG6: TNF-stimulated gene 6
