# Supplementary material for: First trimester human umbilical cord perivascular cells (HUCPVC) modulate the kynurenine pathway and glutamate neurotransmission in an LPS-induced mouse model of neuroinflammation
Source: J Inflamm (Lond). 2023 May 1;20:15. doi: 10.1186/s12950-023-00340-3 (PMC10152638; doi:10.1186/s12950-023-00340-3)
Supplement: Supplementary file 1 — Supplementary Material 1 [file 12950_2023_340_MOESM1_ESM.pdf]

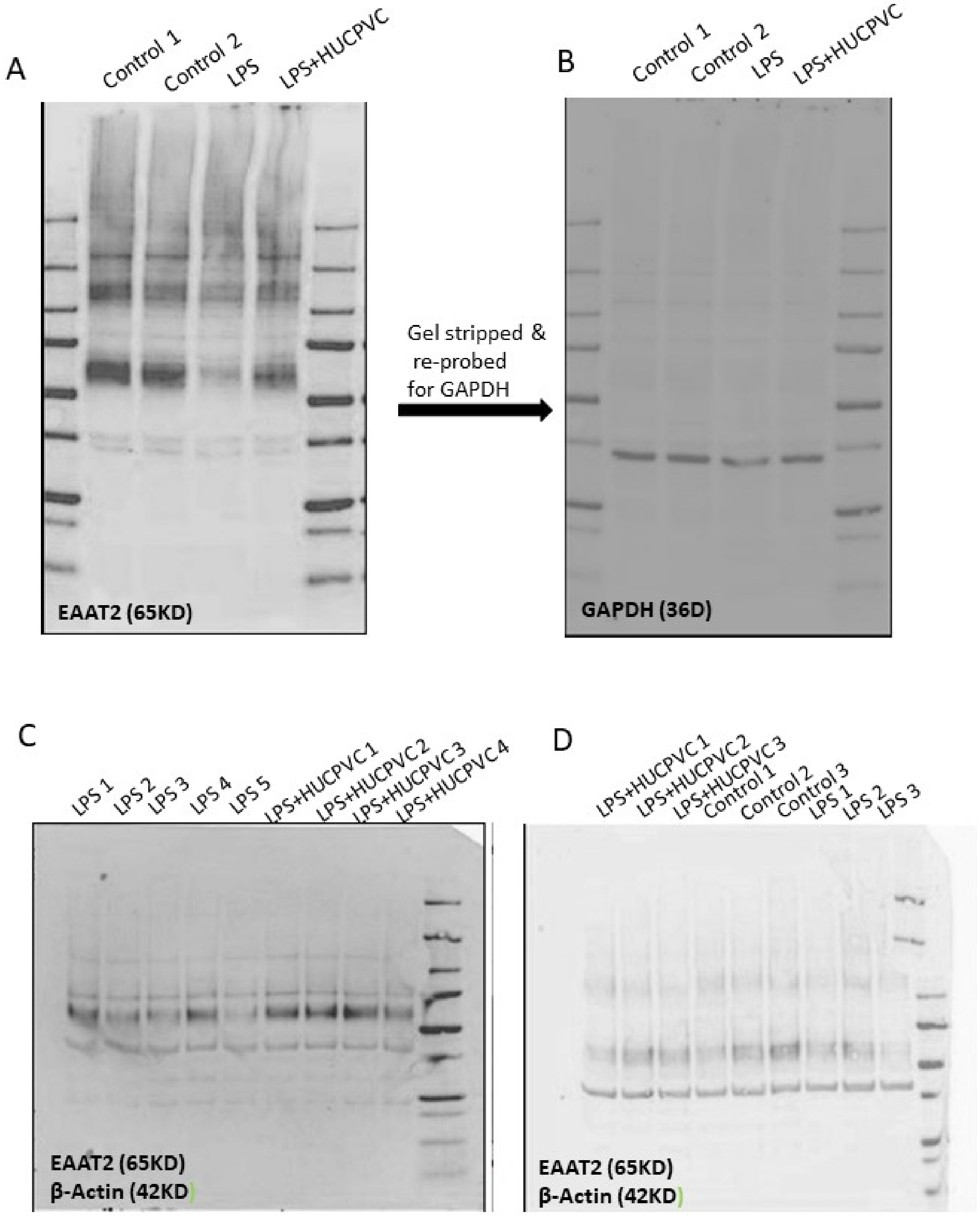

Supplemental Figure 1. Western blot images (A-D) of EAAT2 protein expression in the whole brain homogenate in control, LPS and LPS+HUCPVC groups. GAPDH (B) and β-actin (C,D) were used as loading controls.

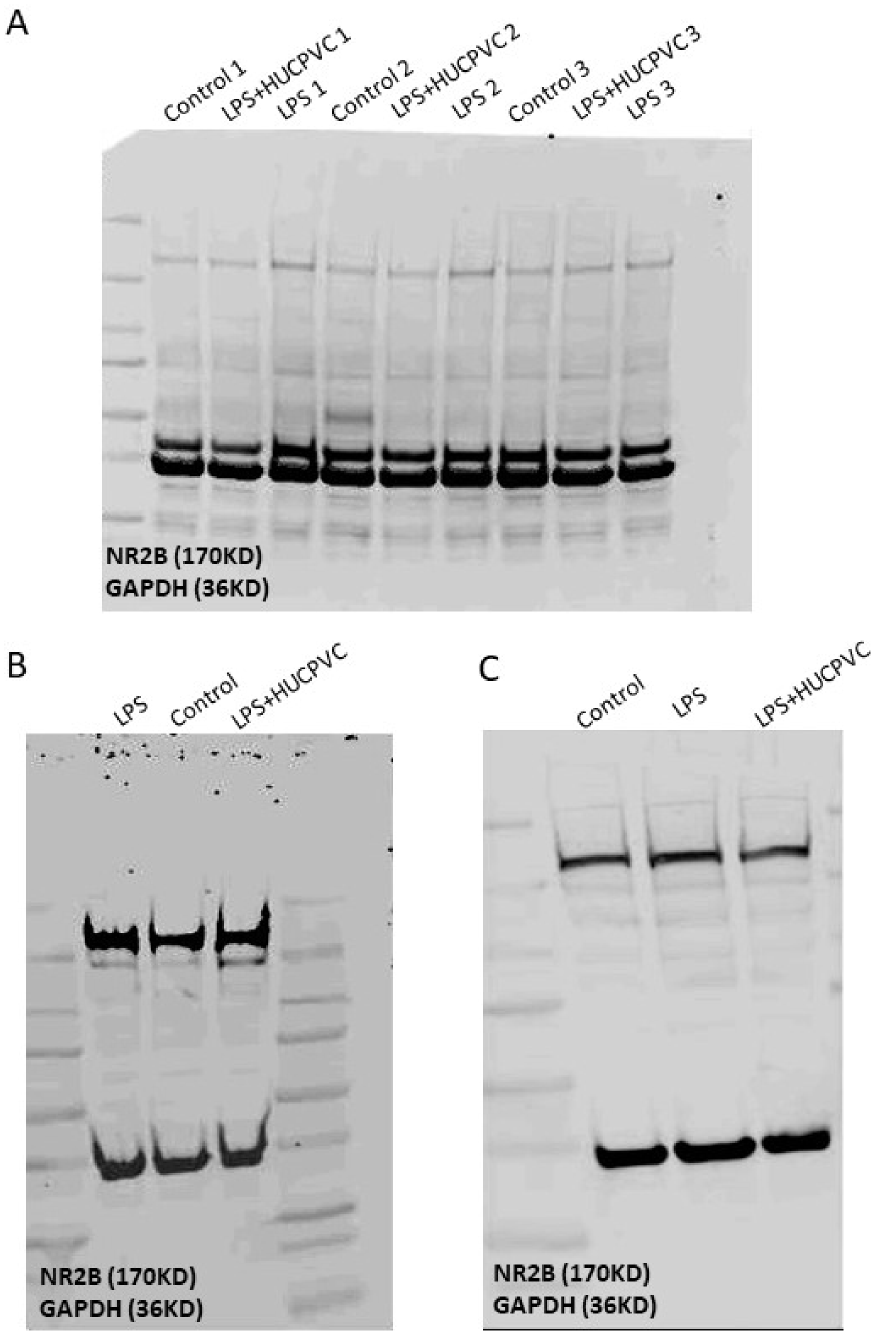

Supplemental Figure 2. Western blot images (A-C) of NR2B protein expression in the synaptosomal fraction in control, LPS and LPS+HUCPVC groups. GAPDH was used as a loading control. (GAPDH bands in 'A' appear white due to overexposure)

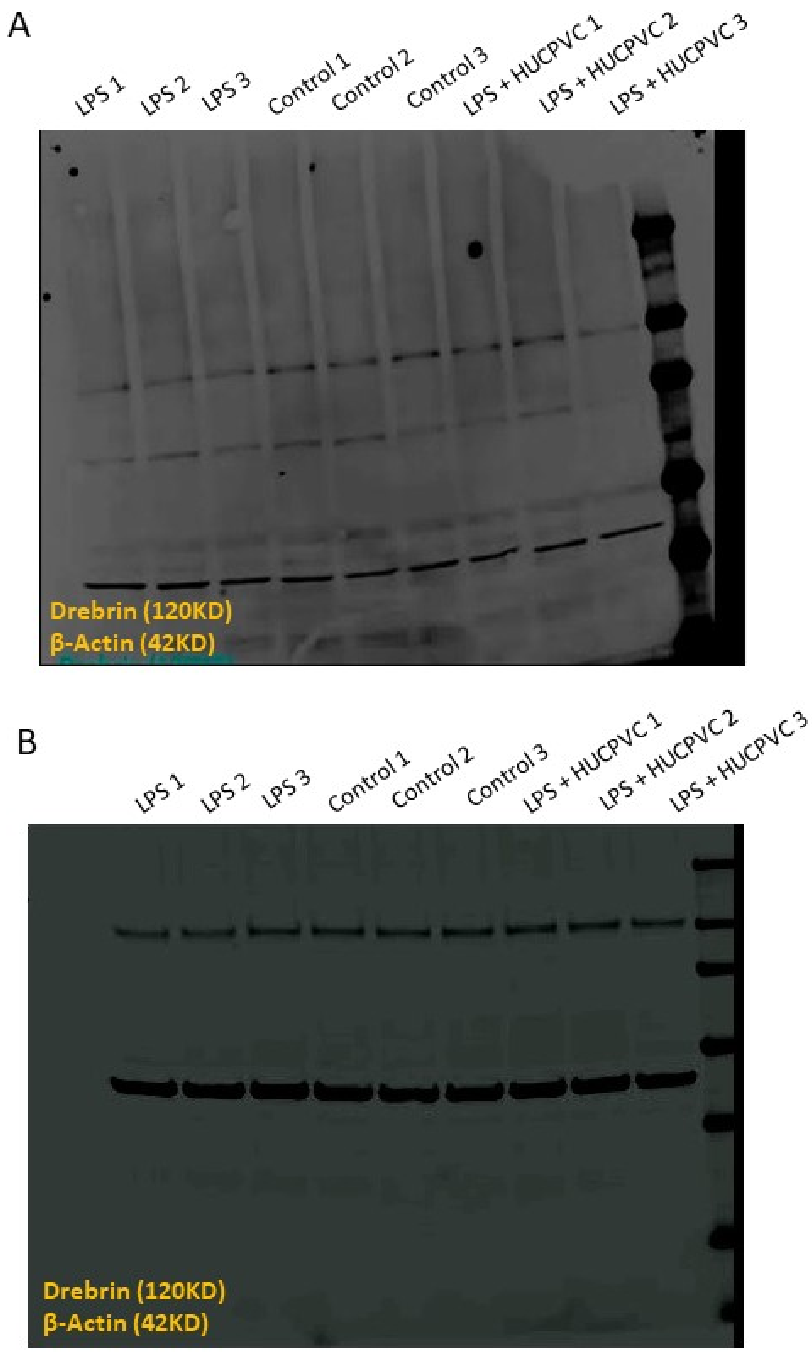

Supplemental Figure 3. Western blot images (A,B) of Drebrin protein expression in the synaptosomal fraction in control, LPS and LPS+HUCPVC groups. β-actin was used as a loading control

A

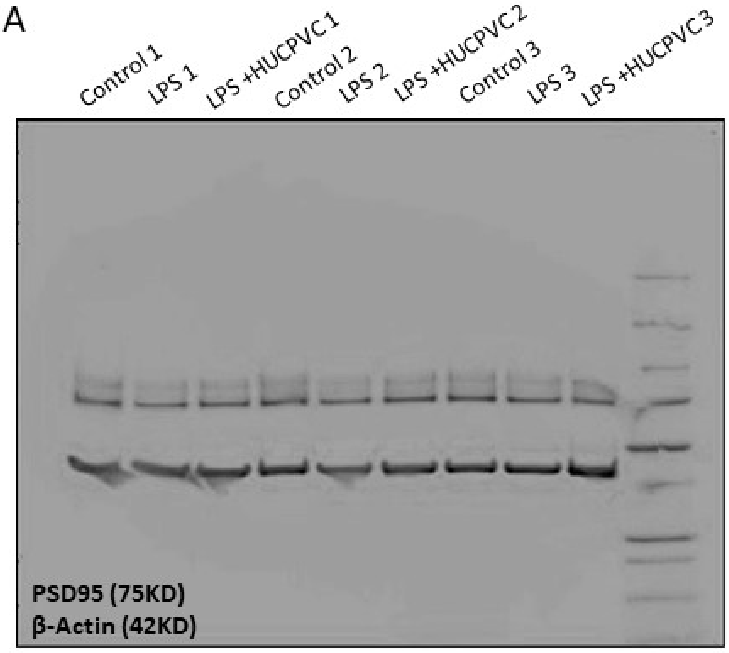

B

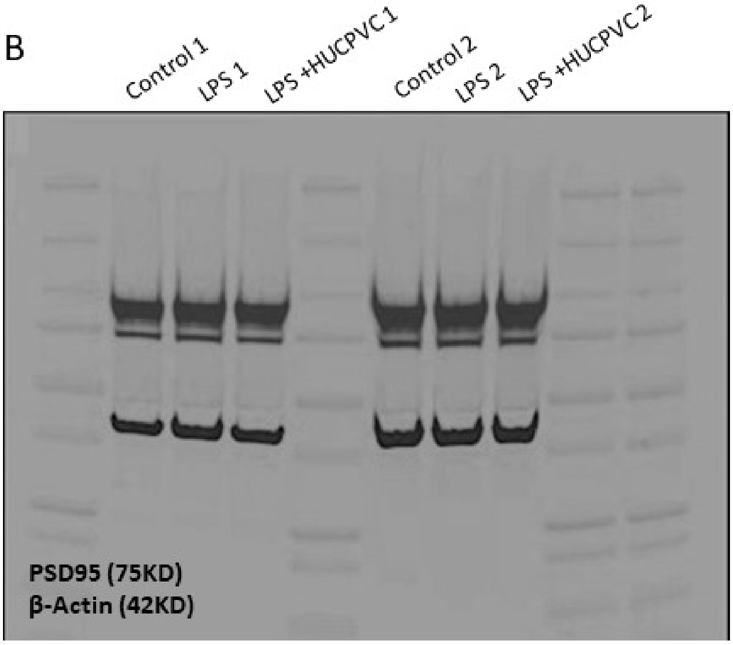

Supplemental Figure 4. Western blot images (A,B) of PSD95 protein expression in the synaptosomal fraction in control, LPS and LPS+HUCPVC groups. β-actin was used as a loading control

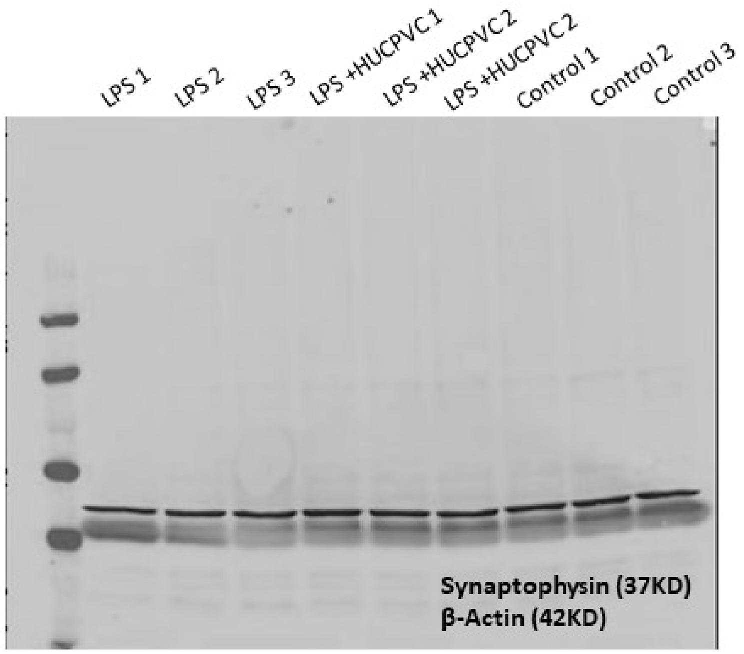

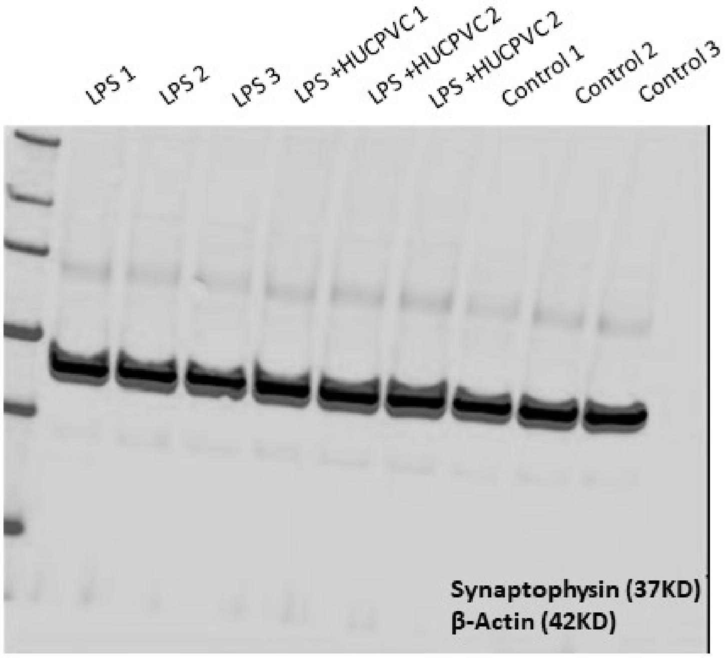

Supplemental Figure 5. Western blot images (A,B) of synaptophysin protein expression in the synaptosomal fraction in control, LPS and LPS+HUCPVC groups. β-actin was used as a loading control
